# Genetic Diversity and Genetic Differentiation of Populations of Golden-Backed Carp (*Cyprinus carpio* var. *Jinbei*) in Traditional Rice Fields in Guizhou, China

**DOI:** 10.3390/ani12111377

**Published:** 2022-05-27

**Authors:** Da Ji, Xin Su, Junjie Yao, Wenzheng Zhang, Rongrong Wang, Shuhai Zhang

**Affiliations:** 1 Research Center of Fishery Resources and Environment, Guizhou University, Guiyang 550000, China; jida314207792@outlook.com (D.J.); shmilu9977@outlook.com (X.S.); wenzheng11241124@outlook.com (W.Z.); wrr17629606186@outlook.com (R.W.); zsh15286385541@outlook.com (S.Z.); 2 Key Laboratory of Animal Genetics, Breeding and Reproduction in the Plateau Mountainous Region, Ministry of Education, Guiyang 550000, China

**Keywords:** 2b-RAD, rice paddies, conservation and breeding, status of germplasm resources

## Abstract

**Simple Summary:**

Rice-fish farming refers to the cultivation of fish in rice fields. China’s Guizhou Province has a long history (over 1000 years) of Rice-fish farming. Carp (golden-backed carp) that have been cultivated for generations in rice fields have evolved to be extremely adaptable to that environment. Unfortunately, farmers and enterprises have found that the feeding ability and growth rate of golden-backed carp are becoming increasingly weaker and slower, respectively. Therefore, the present study carried out to examine the population genetics of golden-backed, and the results indicated that the status of germplasm resources of this species is not very good. The aim of this study was to further elucidate the status of golden-backed carp germplasm resources in this province. In conclusion, this work can provide reliable fundamental data for the future conservation and breeding of golden-backed carp.

**Abstract:**

The aim of this study was to assess the current status of the germplasm resources of golden-backed carp (*Cyprinus carpio* var. *Jinbei*) cultured in paddy fields in Guizhou Province, China. Five populations of golden-backed carp in Liping County, Jinping County, Huangping County, Congjiang County and Duyun City in Guizhou Province were subjected to high-throughput sequencing by 2b-RAD technology, and their genetic diversity and genetic differentiation were analysed. Based on sequencing, 44,896 SNP loci were obtained, and all five population genetic diversity indicators showed low diversity. In the NJ tree, the Congjiang and Liping populations were mixed together, and the other three groups formed a cluster. A cross-validation error box plot and pong cluster plot were constructed to show the K value results. When K = 1, the cross-validation error rate was the lowest. Principal component analysis showed that the Duyun population formed a group separate from the group comprising the other four populations. The genetic differentiation index and genetic distances between the Duyun population and the remaining four populations were greater than 0.05, indicating population differentiation. The genetic diversity of the five populations of golden-backed carp in Guizhou Province was low, the genetic differentiation of the Duyun population was the most significant, and the Duyun population was separate from the other four groups.

## 1. Introduction

Rice-fish farming refers to the cultivation of fish in the water bodies of rice fields. Fish swim in and out of the water in paddy fields, loosening the soil for rice and feeding on insects, mollusks, and plankton, which can greatly reduce pesticide input [1]. Fish manure can serve as a fertiliser for rice, significantly reducing the input of rice fertiliser [2]. Such farming, which can increase the nutritional level, extend the food chain, improve ecological health, and improve the circulation and exchange efficiency of matter and energy, is more conducive to the maintenance and enhancement of system stability than other approaches [3] and can provide substantial economic, ecological, and social benefits [4,5,6]. Rice-fish farming is an organic combination of aquaculture and planting [7] that can support the dual use of a single field for water and double harvests. China was the first country to raise fish in rice fields [8], and Guizhou Province is a traditional Rice-fish farming province in China. In 1975, Eastern Han Dynasty pottery showing a paddy field model was unearthed in Xingyi, Guizhou, China, and proved that Guizhou Province has a history of Rice-fish farming spanning thousands of years [9]. In 2011, the “Congjiang Dongxiang Rice-fish–Duck Composite System” in Guizhou Province was recognised as a globally important agricultural and cultural heritage system by the Food and Agriculture Organization of the United Nations [10].

**Figure 1:**
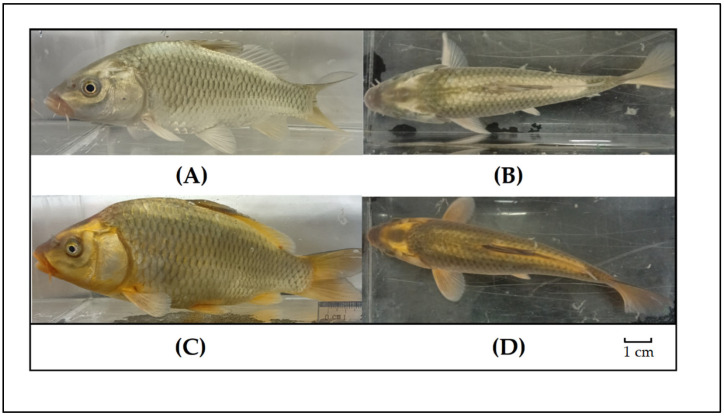
(**A**,**B**) Front and side photos of the LP, HP, JP and CJ populations of the golden-backed carp; (**C**,**D**) front and side photos of the DY population of the golden-backed carp.

The traditional carp cultured for generations in the rice fields of Guizhou Province is known locally as golden-backed carp (*Cyprinus carpio* var. *Jinbei*) (tentative name) because of the two stable golden stripes on both sides of its dorsal fin and a pattern resembling a golden butterfly on its head (Figure 1). The golden-backed carp is mainly distributed in the rice fields of the border areas of Guizhou, Hunan and Guangxi Provinces in China. Long-term artificial and natural selection has made the golden-backed carp highly adapted to rice fields; for example, morphologically, these carp have a red tail with a phosphorus yellow pattern. In terms of living habits, they prefer to remain in rice fields and have a strong foraging ability. They are resistant to hypoxia and temperature differences of more than 20 degrees between day and night and can adapt well to the shallow water environment of rice fields. In recent years, Rice-fish farming has been widely promoted as an ecological farming method in Guizhou Province, where the area of Rice-fish farming reached 1867 × 10^5^ hectares in 2020 [11], and neighbouring provinces. The golden-backed carp, as a very precious indigenous rice field fish germplasm resource in Guizhou, China, has attracted increasing attention.

Biodiversity is the material basis on which human beings depend [12]; however, the current declining level of biodiversity is of widespread concern [13]. Genetic diversity is the foundation and core of biodiversity; the higher the genetic diversity is, the stronger the ability of organisms to adapt to survival, with richer breeding and genetic improvement potential. In the genetic breeding process of aquatic products, genetic diversity is often an important reference index for the selection of breeding materials [14]. Factors influencing genetic diversity can be divided into internal and external causes, with internal causes being the genetic base and external causes being the environmental conditions. The variation in genetic material is the basis for the generation of biological genetic diversity and is closely related to the vitality and reproductive ability of species. In addition, genetic diversity variation in nature is influenced by several factors, including habitat fragmentation and the founder effect [15]. Habitat fragmentation is one of the main causes of biodiversity loss [16]. Habitat fragmentation usually increases the chance of random genetic drift, increases inbreeding rates, and reduces interspecific gene flow, thereby reducing the genetic diversity of plants and animals [17].

The golden-backed carp is a carp variety that has been adapted to paddy fields for a long time. In recent years, an increasing number of farmers and breeding enterprises have found that some golden-backed carp have a weak feeding ability and slow growth rate in paddy fields. The golden-backed carp is a valuable rice field fish in Guizhou, China. The population is small and has been declining; thus, it must be preserved as a genetic resource for future breeding. Before breeding golden-backed carp, it is necessary to understand the current status of its germplasm resources. Genetic diversity and genetic differentiation can reflect the current status of germplasm resources, so it is necessary to study the genetic diversity of golden-backed carp.

In recent years, some scholars have successively studied the genetic diversity and genetic differentiation of carp in rice paddy fields. Ren et al. used microsatellite and direct sequencing technologies to explore nuclear loci and mitochondrial D-loop region and *COI* genes of “paddy field carp” (PF-carp) in Zhejiang Province, China, and they found that the genetic diversity of this population in rice fields was lower than that of the wild population. Through landscape genetic analysis, it was found that farmer behaviour is a key factor affecting the genetic diversity of Qingtian field fish [18]. Gan Baojiang et al. used microsatellites to study the genetic diversity of carp cultured in Guangxi rice fields and found that the average expected heterozygosity, polymorphic information content and observed heterozygosity of rice farming groups were lower than those of wild populations [19]. In earlier research, we combined the mitochondrial D-loop region and Cyt *b* genes and used PCR amplification and a direct sequencing method to study five geographical populations of rice-farm golden-backed carp in Guizhou Province, and we found that the genetic diversity of this carp in different regions of Guizhou ranged from low to high, and that there were different degrees of genetic differentiation [20].

The above studies were limited to two genes, and the polymorphic information content was found to be insufficient, thus reducing the support of high-throughput genome sequencing. Therefore, in this study, 2b-RAD technology was used to perform high-throughput sequencing of 75 individuals in five groups in Guizhou Province, and the genetic diversity and genetic differentiation of the golden-backed carp groups in Guizhou Province were further explored. An in-depth understanding of the characteristics and current status of germplasm resources for golden-backed carp will also provide basic data for the selection and breeding of this fish species.

## 2. Sample Collection and Methods

### 2.1. Materials

**Figure 2:**
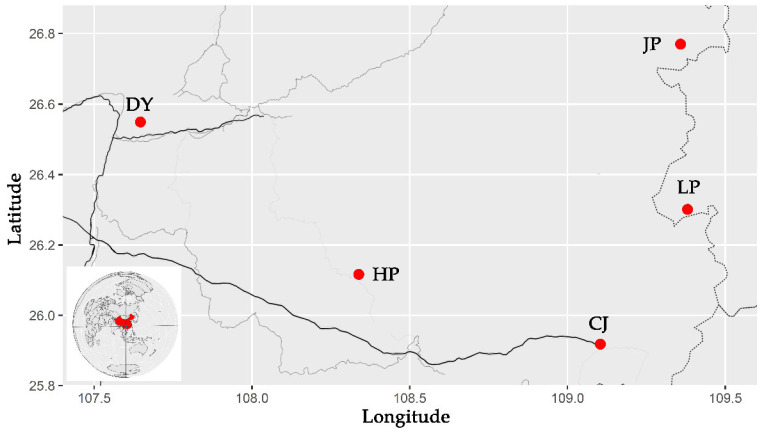
Sampling information diagram (the red circles indicate the sampling points, while uppercase letters indicate the name of the population).

Through a survey, it was found that there are golden-backed carp in five regions of Guizhou Province, China. From August to October 2020, 75 golden-backed carp samples were collected from Jinping County (JP), Liping County (LP), Congjiang County (CJ), Huangping County (HP) and Duyun City (DY) in Guizhou Province (Figure 2). Fifteen individuals were collected at each sampling point (5 paddies were randomly selected from each sampling point, and 3 individuals were randomly selected from each paddy field), and the sampled fish had a body mass of 285.1–451.6 g and a body length of 15.51–22.81 cm.

### 2.2. Extraction and Detection of Genomic DNA

The tail fin of each live golden-backed carp was cut to 0.5 cm^2^, and then the golden-backed carp was returned to the rice field for further breeding. The caudal fin sample was stored in anhydrous ethanol at −20 °C, DNA was extracted using an animal genome extraction kit from Qingke Biotechnology Co., Ltd., Beijing, China, and the obtained DNA samples were analysed by a spectrophotometer to detect the OD values of the samples, which was between 1.8 and 2.0. The samples were stored at −20 °C for later use.

### 2.3. Library Construction and Sequencing

The 2b-RAD libraries were prepared at Qingdao OE Biotech Co., Ltd. (Qingdao, China), as described in Wang [21]. For each sample, 200 ng of genomic DNA was digested using the type IIB restriction enzyme BsaXI, and short adapter sequences were ligated to the ends of the fragments. The ligation products were amplified in 50 µL PCRs with 16 cycles of 98 °C for 5 s, 60 °C for 20 s, and 72 °C for 10 s. The PCR products were purified and recovered with an 8% agarose gel. For each tube, 12 µL of supernatant was used as a template, and the above PCR steps were repeated for 4–6 cycles to improve the yield. PCR products from five samples were mixed, and the mixture was purified using a MinElute PCR Purification Kit. Purification products were digested using SapI (New England Biolabs, Ipswich, MA, USA). The digested product was added to the tube containing pretreated magnetic beads, and the mixture was incubated at room temperature. A magnet was applied, and the supernatant was transferred to a new tube. Then, 200 U of T4 DNA ligase was added to the supernatant, and the mixture was incubated at 16 °C for 45 min. Then, gel purification was performed as described above. Barcodes were introduced by PCR with barcode-bearing primers. PCR products were purified using a MinElute PCR Purification Kit and pooled for sequencing using the Illumina Nova PE150PE platform.

### 2.4. Bioinformatics Analysis

The paired-end reads were merged by Pear software (version 0.9.6) (Creators, Zhang, J.; et al., 2014; Karlsruhe, Germany) [22]. The merged reads were processed using a custom Perl script to trim adaptor sequences. The final 3 nucleotides were also excluded from each read to eliminate artefacts that might have arisen from ligation sites. Reads with more than 8% ambiguous bases (N), of poor quality (15% nucleotide positions with a Phred quality <30), and without restriction sites were removed. The BsaXI tags in the common carp genome (NCBI, https://www.ncbi.nlm.nih.gov/assembly/GCF_000951615.1/, accessed on 8 December 2021) were extracted based on the enzyme’s recognition site, which served as a reference for SNP discovery. High-quality reads of each individual were aligned to the reference genome using SOAP2 (Creators, Li, R.; et al., 2009; Silver Springs, MD, USA) [23] (version 2.21) with the following parameters: r = 0, M = 4, and v = 2. The aligned data for each individual were then used for SNP detection by the RADtyping (Creators, Fu, X.; et al., 2013; Qingdao, China) [24] program with default parameters. For codominant markers, we used a maximum likelihood (ML) algorithm to infer homozygotes and heterozygotes. To obtain robust results in the subsequent analyses, the following criteria were applied for SNP filtering. (1) SNPs that could be genotyped in at least 80% of the individuals were kept for analysis. (2) SNPs with a minor allele frequency (MAF) <0.01 were discarded. (3) Polymorphic loci with more than two alleles possibly derived from sequencing or clustering errors were excluded. (4) Tags with more than two SNPs were excluded. Then, SnpEff (version 4.1g) (Creators, Cingolani, P.; et al., 2012; Detroit, MI, USA) [25] was applied to annotate the filtered SNPs. For genetic analysis, each genotype with markers was assembled head to tail, and missing sites were replaced by “–”.

The analysis of indicators of genetic diversity was performed as follows: the values of expected heterozygosity (He), observed heterozygosity (Ho), polymorphism information content (PIC), and effective number of alleles (Ne) were calculated using a custom Perl script with the following formulas: $He=1-\sum_{i=1}^{k}xi^{2}$, $PIC=1-\sum_{i=1}^{n}P_{i}{}^{2}-\sum_{i=1}^{n-1}\sum_{j=i+1}^{n}2P_{i}{}^{2}P_{j}{}^{2}$ and $Ne=1/\sum_{i=1}^{n}P_{i}{}^{2}$. Ho = the total number of heterozygous individuals observed/the total number of individuals. The Hardy-Weinberg equilibrium *p* value (HW-P) and nucleotide diversity (Pi) were calculated by VCFtools (Creators, Danecek, P.; et al., 2011; Cambridge, UK) [26] software (version 0.1.14).

The genetic differentiation between populations was analysed using the following methods. The population differentiation fixation index (F_ST_) and population genetic distance (DR) were calculated by the R package genepop (version 1.0.5). A neighbour-joining tree was constructed with TreeBeST (Creators, Vilella, A.J.; et al., 2009; Cambridge, UK) [27] (version 1.9.2) under the p-distances model with bootstrapping (1000). The R package ggtree (v1.16.6) was used to visualise the phylogenetic tree. To determine the optimal number of populations (K) and population structure, the SNP genotyping information was analysed using ADMIXTURE (version 1.3.0) (Creators, Alexander, D.H.; et al., 2009; Los Angeles, CA, USA) [28]. Principal component analysis (PCA) of the SNPs was performed using plink2 (version 2.0) (Creators, Purcell, S.; et al., 2007; Boston, MA, USA) [29].

## 3. Results

### 3.1. Genetic Diversity Parameters of Five Geographical Populations of Golden-Backed Carp in Guizhou, China

Based on the selection of 75 individuals in five populations of golden-backed carp, 44,896 SNPs were finally screened for genetic diversity analysis, and the results are shown in Table 1. In the five populations, the HW-P ranged from 0.882 to 0.900, and the average for the five groups was 0.890. The expected degree of heterozygosity (He) ranged from 0.112 to 0.126, with an average of 0.119. The observed degree of heterozygosity (Ho) ranged from 0.116 to 0.139, with an average of 0.127. The PIC ranged from 0.095 to 0.108, with an average of 0.100. The number of effective alleles (Ne) ranged from 1.168 to 1.188, with an average of 1.179. The nucleotide diversity (Pi) ranged from 0.116 to 0.131, with an average of 0.123. All genetic diversity indicators were highest in the LP population and lowest in the HP population.

### 3.2. Genetic Differentiation of Five Populations of Golden-Backed Carp in Guizhou, China

**Figure 3:**
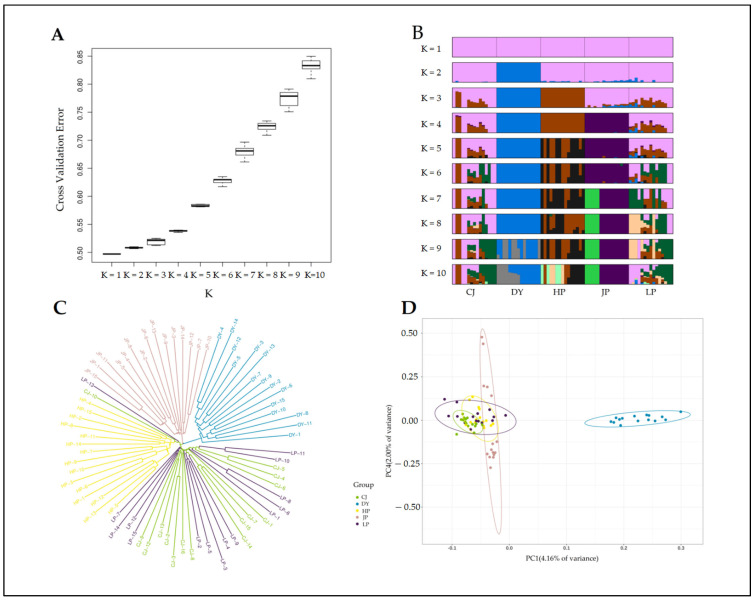
Genetic structure of five populations of golden-backed carp in Guizhou, China. CJ, DY, HP, JP and LP denote the Congjiang, Duyun, Huangping, Jinping and Liping populations, respectively. (**A**) A cross-validation error box plot. (The abscissa takes different K values, which indicate the number of hypothetical populations, while the ordinate represents the cross-validation error value. The cross-validation error of K values ranges from 1 to 10; generally, the lowest CV value is the optimal K value, but the results of other analyses need to be considered). (**B**) Based on the 5 populations of golden-backed carp, pong clustering was performed for multiple replicated results of each K value from 1 to 10 (each individual is represented by a single vertical line divided into K colours, where K is the number of clusters assumed, and different colours represent different populations. Black lines separate individual populations whose names are indicated below the diagram). (**C**) Neighbour-joining tree of 75 individuals in the 5 populations based on 44,896 SNPs. (**D**) Principal component analysis (PCA) was performed based on the degree of SNP differentiation in individual genes (PC1 and PC2 with the largest SNP variance were selected as the horizontal and vertical axes for PCA).

Based on the 44,896 SNP loci screened, the genetic structure of five golden-backed carp populations was analysed. The cross-validation error results showed that when K = 1 (identifying five populations as one population), the error rate was the lowest, and the error rate increased as the K value increased (Figure 3A). At the same time, we performed a pong cluster analysis of values from K = 1 to K = 10, and the enhanced plots also showed that when K = 1, the colours appeared pink and mixed with other colours. When K = 2, the large group formed a single blue region, mixed with only a few colours from the other four groups. From K = 3 to 10, the degree of colour confluence increased with an increasing K value (Figure 3B).

The neighbour-joining tree showed that the LP population and the CJ population were mixed together and formed a single branch, and the HP group was mixed with an individual from the CJ and LP populations. In addition, the DY and JP populations each clustered independently into a branch (Figure 3C). The PCA results were inconsistent with the above results (Figure 3D), and the blue dots representing the DY population were independently clustered into an ellipse, which was located the farthest from the remaining four populations and did not intersect with the dots for the other four populations. The JP, LP, and CJ populations were closely intermingled. The HP population was located relatively far from these three populations, but there was still some overlap.

The genetic differentiation index and genetic distance between the populations were between 0.007 and 0.094 and 0.007 and 0.099, respectively (Table 2). The genetic differentiation index of the DY population and the four populations HP, JP, LP and CJ was the largest, with values of 0.094, 0.094, 0.075 and 0.084, respectively, and the genetic distance was also the farthest, at 0.098, 0.099, 0.078 and 0.087, respectively. In summary, by combining the NJ tree, cross-validation error box plot, pong cluster plot and PCA results, we believe that the DY population and the remaining four populations can be considered two populations. 

## 4. Discussion

### 4.1. The Current Status and Reasons for the Low Genetic Diversity of Golden-Backed Carp in Five Geographical Populations in Guizhou, China

Monitoring the genetic diversity and genetic structure of small biological populations is crucial for protecting their genetic diversity and the uniqueness of the gene pool [30]. Golden-backed carp cultivation is ongoing, but basic research and reports on their genetic breeding are very scarce; therefore, we studied the current state of genetic diversity. Judging from genetic diversity indicators such as PIC, Ho, and He, the genetic diversity of the five rice-farm golden-backed carp populations in Guizhou was low. For example, the PIC is an important indicator of the degree of polymorphism at gene loci in a population and can reflect the degree of genetic variation of the population. According to Botstein D. et al. [31], when the PIC is less than 0.25, the polymorphism is low. The PIC values of the five golden-backed carp populations showed low polymorphism.

The mean heterozygosity of the population is a common parameter for measuring the genetic variation in a species, and the higher the average heterozygosity of the population is, the lower the genetic consistency of the population, the higher the genetic variation, and the richer the genetic diversity [32]. The expected heterozygosity (He) and observed heterozygosity (Ho) of the five golden-backed carp populations in this study averaged 0.119 and 0.127, respectively, which were much lower than the threshold value of 0.3 (which can distinguish one population from others based on genetic diversity) [33], which also indicated extremely low genetic diversity in the golden-backed carp. It is worth mentioning that although genetic diversity has a universal threshold, many factors can affect the genetic diversity indicators we detect, such as the type of marker, the method of molecular labelling, study systems, and studied organisms. Notably, the expected degree of heterozygosity (He) of the five populations was lower than the observed degree of heterozygosity (Ho). A study conducted by Sophie showed that the closer the Hardy–Weinberg equilibrium *p* value (d) is to 0, the closer the genotype distribution is to equilibrium, while d > 0 indicates excess heterozygosity [34]. Based on this rule, the five populations in the present study exhibited excess heterozygosity, as their Hardy–Weinberg equilibrium *p* values ranged from 0.882 to 0.900. Related studies have shown that if a population bottleneck occurs, the population size decreases, and the degree of heterozygosity will be excessive [35]. Thus, a bottleneck event may have occurred in the population of golden-backed carp.

Habitat fragmentation is one of the most important factors affecting the genetic diversity of animals and plants [36]. In traditional agriculture-based economies, Rice-fish farming is widely distributed, and the culture area is highly contiguous. With the advancement of aquatic intensification, Rice-fish farming has gradually been replaced by intensive farming with high production efficiency, and the breeding area has gradually been transformed into small populations. The significant reduction in genetic exchange between golden-backed carp populations in rice paddies is one important reason for the low genetic diversity of the species. On the other hand, according to the genetic drift results (F; F = 1/2 Ne), the Ne values in the five populations of golden-backed carp in this study were less than 2, indicating that the fish underwent strong genetic drift. Genetic drift is inversely proportional to population size [15], and the population of golden-backed carp is shrinking, which aggravates genetic drift, increases the likelihood of alleles being lost, and leads to low genetic diversity.

In addition, due to habitat fragmentation and population reduction, an increasing number of local farmers need to purchase fry from external sources, which continues to trigger a founder effect. The founder effect refers to the fact that when a small number of individuals or fertilised mothers in a large population move into a new environment to establish a new population, they often carry only a small part of the gene pool of the parent population, and the gradual loss of genetic diversity is caused by a series of founder events that occur as a result of the group entering a previously uninhabited area [37]. Therefore, a founder effect may also be an important reason for the overall low genetic diversity of the five populations of golden-backed carp.

Biodiversity is closely related to environmental differences, and the greater the pressure of natural selection is, the richer the diversity [38]. In Guizhou Province, the 1000-year history of paddy fields being used for cultivation has caused golden-backed carp to be highly adapted to paddy fields, and there are no natural enemies of carp in the water, which provides long-term stable habitats for generations. Because natural selection pressure is low, mutation rates are low, which is another important reason for the low genetic diversity of the golden-backed carp population.

### 4.2. Status Quo of Genetic Differentiation among Populations of Golden-Backed Carp in Guizhou, China

The degree of genetic differentiation between the DY population and the remaining populations was the greatest. Quan YC et al. proposed the following criteria for genetic differentiation between populations: 0 < F_ST_ < 0.05 reflects a low level of equal differentiation, and 0.05 < F_ST_ < 0.15 reflects a moderate differentiation level [39]. The genetic differentiation index (F_ST_) of the DY population and the remaining four populations was greater than 0.05. Genetic distances also showed that the genetic differentiation of the DY population was the greatest. Genetic distance was calculated at the genus, species, and population levels according to Shaklee et al. [40]. The genetic distances at these three levels were 0.9, 0.3 and 0.05, respectively. Similarly, the genetic distance of the DY population from the remaining four populations was greater than 0.05, indicating a moderate level of differentiation.

The K value cross-validation error box plot and the pong cluster plot showed that when K = 1, the cross-validation error rate was the lowest. By combining the genetic differentiation coefficients (F_ST_), genetic distance (DR), neighbour-joining tree and PCA results, we believe that the DY population has developed more obvious genetic differentiation from the remaining four populations. In our research, we found that compared with other populations, the DY population was extremely small. At present, the golden-backed carp is cultivated at a small scale in only one village, resulting in long-term inbreeding in the DY population. Therefore, inbreeding and a reduction in the number of breeders are the key factors leading to population differentiation. This is similar to the findings of Santos S. [41]. Each fragmented population will have different fixed alleles, which will cause the populations to form different clusters in PCA. Instead, the populations examined here clustered together (except for the DY population), suggesting that they shared a common ancestor. They all probably came from the same founding population (possibly excluding DY), and this population was very small.

Compared with the other four populations, the DY population also showed obvious differentiation in morphology. The body colour of the DY population was dark red (Figure 1C,D), while that of the other four populations was black. In addition, the remaining four populations of golden-backed carp also have a certain degree of differentiation in body colour, with different shades of blackness, some shallow, and some dark. Population isolation is an important factor influencing genetic differentiation [42], and the current separation of rice field breeding areas of golden-backed carp has increased the population isolation, resulting in a great reduction in gene exchange between various groups of golden-backed carp and different degrees of genetic differentiation.

### 4.3. Recommendations for the Conservation of Germplasm Resources of Golden-Backed Carp

According to existing reports, golden-backed carp are distributed only in the border areas of Guizhou, Guangxi and Hunan Provinces in China, and the current distribution range and population size are very small. Rice field fish farming is a green, sustainable method of ecological breeding, and golden-backed carp have good rice field adaptability. They are a precious rice field fish germplasm resource. The genetic diversity of the current golden-backed carp population is very low, and production has shown a slow growth rate, body colour changes, and other disadvantages. The selection and protection of golden-backed carp is important; therefore, the following recommendations are made:

1. Purify the germplasm of the golden-backed carp and use pure-breeding parents for body colour for artificial reproduction.
2. For the restoration of the germplasm of the golden-backed carp, the scope of breeding should be expanded as much as possible, inbreeding should be avoided, and good seeds should be regularly introduced from foreign breeding farms as breeding parents to improve the genetic diversity of offspring.
3. Make full use of the globally important agricultural cultural heritage of the Congjiang Dongxiang Rice-fish-Duck Composite System area to carry out protection work.

## 5. Conclusions

In summary, we used 2b-RAD technology to molecularly label the genomes of five existing rice-reared golden-backed carp populations in Guizhou Province, China. Based on the screening of 45,789 SNP loci, we found that the genetic diversity level of the rice-farmed golden-backed carp population in Guizhou Province was very low, except for the DY population. The degree of interpopulation differentiation was very small, and the protection of golden-backed carp germplasm resources is important. This study could provide basic data for future breeding of golden-backed carp in paddy fields.

## Figures and Tables

**Table 1:** Genetic diversity parameters of five populations of golden-backed carp in Guizhou, China, based on 44,896 SNPs.

Group	HW-P	He	Ho	PIC	Ne	Pi
CJ	0.900	0.123	0.137	0.104	1.183	0.127
DY	0.883	0.115	0.118	0.096	1.177	0.119
HP	0.887	0.112	0.116	0.095	1.168	0.116
JP	0.882	0.117	0.123	0.099	1.179	0.122
LP	0.896	0.126	0.139	0.108	1.188	0.131

Note: CJ, DY, HP, JP and LP denote the Congjiang, Duyun, Huangping, Jinping and Liping populations, respectively.

**Table 2:** Table of interpopulation genetic differentiation coefficients (F_ST_) and genetic distance (DR).

Group	DY	HP	JP	LP	CJ
DY		0.098	0.099	0.078	0.087
HP	0.094		0.075	0.050	0.053
JP	0.094	0.073		0.055	0.059
LP	0.075	0.049	0.054		0.007
CJ	0.084	0.052	0.057	0.007	

Note: CJ, DY, HP, JP and LP denote the Congjiang, Duyun, Huangping, Jinping and Liping populations, respectively. The lower triangle is the interpopulation genetic differentiation coefficient (F_ST_), and the upper triangle is the interpopulation genetic distance (DR).

## Data Availability

Not applicable.
